# An Experimental Study on the Performance Evaluation and Thermodynamic Modeling of a Thermoelectric Cooler Combined with Two Heatsinks

**DOI:** 10.1038/s41598-019-56672-9

**Published:** 2019-12-30

**Authors:** Marzieh Siahmargoi, Nader Rahbar, Hadi Kargarsharifabad, Seyed Esmaeil Sadati, Amin Asadi

**Affiliations:** 1 Department of Mechanical Engineering, Semnan Branch, Islamic Azad University, Semnan, Iran; 2 Young Researchers and Elite Club, Semnan Branch, Islamic Azad University, Semnan, Iran; 3 Energy and Sustainable Development Research Center, Semnan Branch, Islamic Azad University, Semnan, Iran; 4 Division of Computational Physics, Institute for Computational Science, Ton Duc Thang University, Ho Chi Minh City, Vietnam; 5 Faculty of Electrical and Electronics Engineering, Ton Duc Thang University, Ho Chi Minh City, Vietnam

**Keywords:** Mechanical engineering, Thermoelectric devices and materials

## Abstract

The present study aims to investigate the performance of a one-stage thermoelectric cooler using mathematical and thermodynamic modeling and proposing a new correlation for performance evaluation of a thermoelectric cooler combined with two heatsinks. Validating the results of the proposed correlation, a series of experiments have been carried out on the same system. The system consists of a thermoelectric cooler and a heatsink on each side. Deriving the governing equations of the system, the effects of changing the voltage and the thermal resistance of the cold- and hot-side heatsink on cooling power, the cold-side temperature of thermoelectric, and the coefficient of performance of the system have been investigated. The results indicated that changes in voltage have a considerable effect on the performance of the system. Moreover, the maximum cooling power of the system takes place at the voltage of 14 *V*, which is the optimum voltage of the system. Furthermore, decreasing the thermal resistance of the hot-side heatsink leads to increasing the cooling power and the cold-side temperature of the thermoelectric cooler. On the other hand, increasing the thermal resistance of the cold-side heatsink leads to decreasing the cooling power of the system.

## Introduction

In recent years, due to the diverse applications of thermoelectric (TE) modules (cooler and generator), they have been widely used by researchers in different applications^1–4^ such as Cai *et al*.^5^ investigated the energy efficiency and entropy generation minimization of two thermoelectric systems for cooling of electronics, Manikandan *et al*.^6^ studied the pulse operation of a thermoelectric cooler (TEC) in the application of building cooling, Lekbir *et al*.^7^ proposed a new configuration of a waste heat recovery system for a concentrated photovoltaic system employing thermoelectric generators (TEG) and nanofluids and so forth^8–10^. Thermoelectric (TE) modules have several advantages, such as having no moving parts, compact size, they do not need any maintenance, having the capacity of cooling under ambient temperature, and so forth. During the past decade, there has been an increasing amount of literature on different aspects and applications of TEs^11–15^.

Many experimental and analytical investigations have been conducted to study different aspects of TECs and the effects of various parameters on the performance and cooling power of them. Esfahani *et al*.^16^, Rahbar and Esfahani^17^, and Rahbar *et al*.^18^ conducted some experimental investigations on the effects of using TECs in the cooling system of three different types of solar stills and investigated the performance and productivity of those systems. Hu *et al*.^19^ experimentally investigated the effect of using TEC on a central processing unit (CPU) under severe environmental condition. They studied the performance of the TEC at two different temperatures and operating conditions. Dai *et al*.^20^ experimentally investigated the performance of a solar-driven thermoelectric refrigerator. They reported that the COP of the system was about 0.3 which strongly depends on solar radiation and the temperature difference between two sides of thermoelectric modules. Sungkar *et al*.^21^ used a combination of a thermoelectric cooler and heat pipes to make a reliable refrigeration system. The optimum COP of their system was 0.182 when the refrigerator operated at an input power of 40 W. He *et al*.^22^ experimentally investigated the performance of a thermoelectric cooling and heating device driven by a heat pipe photovoltaic/thermal (PV/T) panel. The results showed that the energy efficiency of the system in summer operation mode is higher than that of winter operation mode. The COP of the system was reported as about 1.7 with a thermal efficiency of 23.5%.

In addition to experimental investigations, there is a large volume of published studies using mathematical modeling to describe the role of TECs in various industrial and engineering applications. In this regard, an analytical study has been performed on evaluating and optimizing the performance of a TEC by Zhang^23^. The effect of hot- and cold-side temperature difference has been examined in this study. In another numerical investigation, the performance of miniature TEC affected by the Thomson effect has been studied by Chen *et al*.^24^. They have analyzed three different TECs in order to investigate the performance of miniature TECs through a three-dimensional numerical simulation. Their results showed that increasing the number of pairs of TEC leads to a noticeable increase in the cooling power of the module. The role of surface radiation on the performance of TEC with heat sink has been investigated by Sarkar and Mahapatra^25^. They have validated the results of modeling with those provided by the company fabricated the heat sink and TEC. Dehghan *et al*.^26^ conducted thermal modeling of a thermoelectric assisted solar still. They used a TEC to examine the temperature difference between the evaporating and condensing areas. Manikandan and Kaushik^27^ analytically investigated the performance of an annular thermoelectric cooler. They derived new expressions for the optimum current at the maximum energy/exergy efficiency and maximum cooling power conditions. They showed that the cooling power, energy, and exergy efficiency of the system is lower than flat plate thermoelectric coolers. There is also other literature that investigated different aspects of thermoelectric modules under different operating conditions^28–32^.

Based on what has been discussed, it can be concluded that the cooling power of a thermoelectric module is one of the most important parameters in cooling applications. This is directly depending on the internal thermoelectric parameters, supplied voltage, surrounding temperature, and the thermal resistance of both sides of the thermoelectric module. To the best of the authors’ knowledge, there is no comprehensive study in the literature on the effects of changing voltage and thermal resistant of the heatsinks on the performance of thermoelectric modules. This study aims to investigate the effect of changing the thermal resistance of two heatsinks located on both sides of a thermoelectric cooling module on the cooling performance of the system. Moreover, it is tired to propose a new correlation for performance evaluation of a thermoelectric cooler combined with two heatsinks. The results would assist the designers and engineers in working with a system of thermoelectric cooler and heatsinks; a good example of a practical application would be the thermoelectric-based refrigerators. Furthermore, the proposed method would ease the decision-making process in selecting the best value of voltage and system characteristics in a given ambient temperature and heatsinks’ thermal resistance.

## Theoretical Background

**Figure 1:**
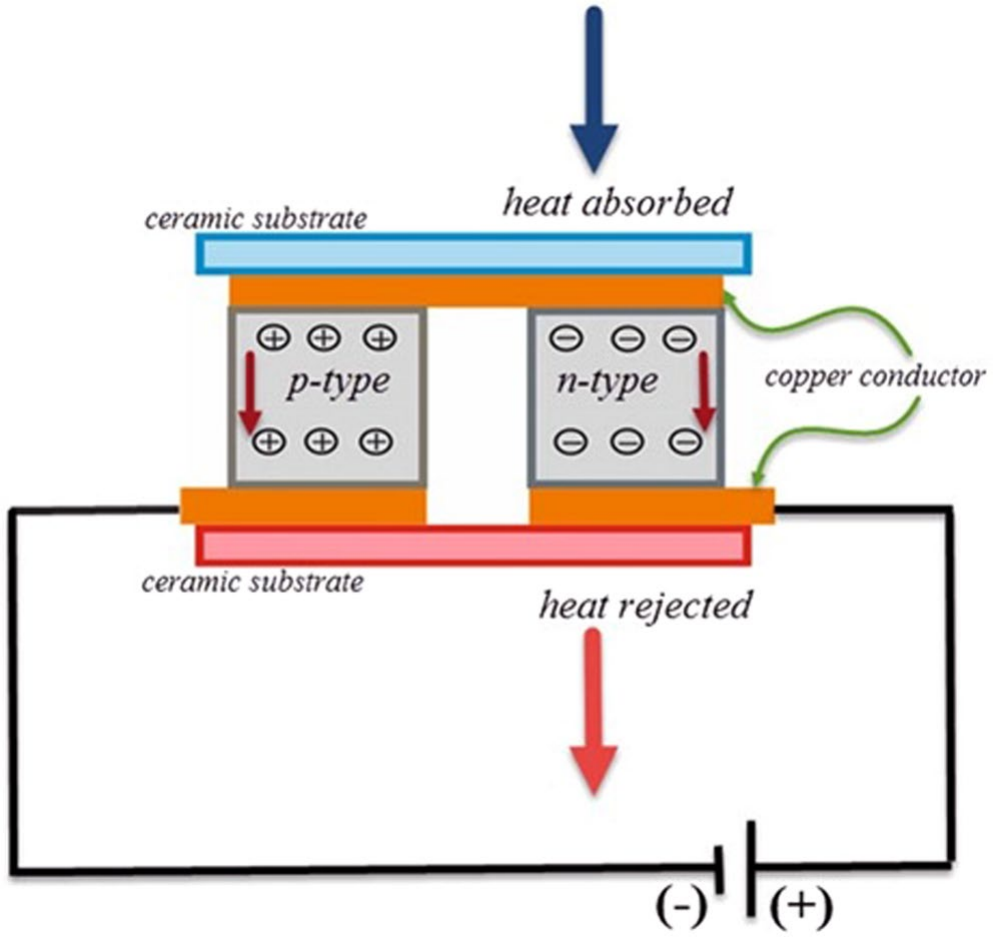
A schematic view of a thermoelectric cooler.

Literally, thermoelectrics is associated with electrical and thermal phenomena. They can convert electrical energy into thermal energy and vice versa. There are two types of thermoelectric modules; thermoelectric cooler and thermoelectric generators. It is known that there is a great potential in utilizing thermoelectric generators in waste heat recovery applications while thermoelectric coolers have great potential in providing refrigeration and temperature control in broad applications such as electronic packaging and medical devices^33^. In a thermoelectric cooler, a temperature difference is produced between two sides of the module by supplying direct current. Figure 1 shows a schematic view of a thermoelectric cooler and the electrical circuit. It can be seen that heat is absorbed from the cold-side of TEC, and it is transferred to ambient from the hot-side of the thermoelectric module.

The input and output heat from two sides of a thermoelectric cooler can be calculated as follows^34–37^:

1 $${Q}_{c}=\alpha I{T}_{c}-0.5\beta {I}^{2}-\gamma \Delta T$$

2 $${Q}_{h}=\alpha I{T}_{h}+0.5\beta {I}^{2}-\gamma \Delta T$$

where ***α***, ***β***, ***γ***, ***I***, ***Q***_***c***_, ***Q***_***h***_, ***Tc***, ***T***_***h***_ and ***∆T*** represent the Seebeck coefficient, electrical resistance, thermal conductance, electrical current, heat rate at the cold side (*W*), heta rate at the hot side (*W*) cold surface temperature (*K*), hot surface temperature (*K*) and the temperature difference between the cold and hot sides (*K*) through a thermoelectric module, respectively. The temperature difference between the two sides of a TEC can be express as follows:

3 $$\varDelta T={T}_{h}-{T}_{c}$$

The difference between the input and output heat from two sides of a TEC is equal to the supplied power. The supplied power and voltage can be calculated by the following equations:

4 $$V=\alpha \varDelta T+\beta I$$

5 $$W=VI$$

where ***W*** is the power supply (*W*) and ***V*** represents voltage (*V*). The coefficient of performance (COP) of a cooling system is the ratio of cooling power per supplied power, and it is expressed as follows:

6 $$COP=\frac{{Q}_{c}}{W}$$

Internal parameters of thermoelectric modules are private, and manufacturers of TECs do not express them in the catalog of thermoelectric modules. Palacios^34^ reported a method to estimate internal thermoelectric parameters as follows:

Substituting ∆T = 0 in Eq. (4), the electrical resistance can be calculated as follows:

7 $$\beta =\frac{V}{I}$$

On the other hand, the manufacturers provide performance curves in different conditions in the datasheet of commercial thermoelectric modules. Extracting the values of ***I*** and ***V*** from the performance curve and substituting in Eq. 7, the electrical resistance can be calculated. It was proposed that in order to decrease the estimation error, different values of electrical current and voltage should be selected, and their average values should be used to calculate the electrical resistance. Substituting ***β*** in Eq. (1), the Seebeck coefficient (***α***) can be calculated as follows:

8 $$\alpha =\frac{{Q}_{c}+0.5\beta {I}^{2}}{I{T}_{c}}$$

Moreover, in order to calculate the thermal conductance (***γ***), substituting ***Q***_***c***_ = 0 in Eq. (1), ***γ*** can be calculated for each ***I*** and ***∆T*** as follows:

9 $$\gamma =\frac{\alpha I{T}_{c}-0.5\beta {I}^{2}}{\varDelta T}$$

## Mathematical Modeling Formulation

**Figure 2:**
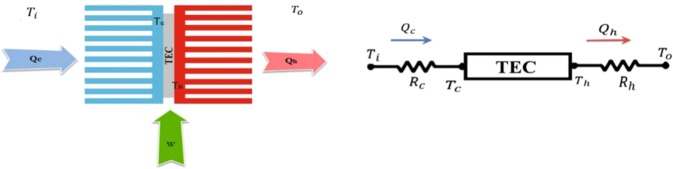
A schematic view of the studied system and its thermal resistance network.

In this paper, a single thermoelectric cooling system has been studied. The system consists of a thermoelectric cooler module and two heatsinks mounted on each side of the TEC. Nowadays, heatsinks are the most well-known devices for augmentation of heat transfer from a hot surface. Without using such a device, the TEC is vulnerable to excess heating, and if the temperature of the hot-side reaches the melting point of solders, the thermoelectric module will fail. Figure 2 shows a schematic view of the studied system and its thermal resistance network. In this figure, ***Q***_***c***_ is the cooling power of a TEC, ***Q***_***h***_ represents the exit heat from a TEC, and ***W*** is the supplied electrical power to the system.

The absorbed heat by the cold-side and the transferred heat from the hot-side of the heat sink can be written as follows^38,39^:

10 $${Q}_{c}=\frac{{T}_{i}-{T}_{c}}{{R}_{c}}$$

11 $${Q}_{h}=\frac{{T}_{h}-{T}_{o}}{{R}_{h}}$$

where ***T***_***i***_, ***T***_***c***_, ***T***_***h***_, ***T***_***o***_, ***R***_***c***_, and ***R***_***h***_ are the cold-side ambient temperature, the cold- and hot-side temperature of TEC, and thermal resistance of the cold- and hot-side heat sinks, respectively.

Combining the Eqs. (1) and (10), ***T***_***c***_ can be calculated as follows:

12 $${T}_{c}=\frac{{T}_{i}+0.5\beta {I}^{2}{R}_{c}+\gamma \varDelta T{R}_{c}}{\alpha I{R}_{c}+1}$$

Likewise, ***T***_***h***_ can be calculated by combining the Eqs. (2) and (11):

13 $${T}_{h}=\frac{{T}_{o}-0.5\beta {I}^{2}{R}_{h}+\gamma \varDelta T{R}_{h}}{1-\alpha I{R}_{h}}$$

Using Eqs. (3), (12) and (13), ***∆T*** can be expressed as follows:

14 $$\varDelta T=\frac{0.5\beta {I}^{2}({R}_{h}-{R}_{c})-\alpha I{R}_{c}({T}_{o}-{T}_{i})}{{\alpha }^{2}{I}^{2}{R}_{h}{R}_{c}+\alpha I({R}_{h}-{R}_{c})+\gamma ({R}_{h}+{R}_{c})}$$

On the other hand, with respect to Eq. (4) ***∆T*** can be calculated as follows:

15 $$\varDelta T=\frac{V-\beta I}{\alpha }$$

Combining the Eqs. (14) and (15), a third-order nonlinear equation to calculate the supplied electrical current is derived as follows:

16 $$\begin{array}{c}({\alpha }^{2}\beta {R}_{c}{R}_{h}){I}^{3}-({\alpha }^{2}{R}_{c}{R}_{h}V-\alpha \beta ({R}_{h}-{R}_{c})-0.5\beta \alpha ({R}_{h}+{R}_{c}))\\ {I}^{2}-({\alpha }^{2}{R}_{c}({T}_{o}+{T}_{i})-\beta \gamma ({R}_{h}+{R}_{c})+\beta +\alpha V({R}_{h}-{R}_{c}))\\ I-(\gamma ({R}_{h}+{R}_{c})-1)V=0\end{array}$$

where ***R***_***c***_ and ***R***_***h***_ represents resistance of the heat sink at the cold and hot surface (*KW*^*−1*^), respectively. Equation (16) is a general nonlinear correlation for a thermoelectric cooling system combined with two heatsinks. This equation is a function of internal thermoelectric properties, cold ambient temperature (***T***_***i***_), hot ambient temperature (***T***_***o***_), the thermal resistance of the cold-side heatsink (***R***_***c***_), the thermal resistance of the hot-side heatsink (***R***_***h***_), and supplied electrical voltage (***V***). Solving the above equation, the supplied electrical current will be calculated. Having an electrical current and using Eqs. (1–5) and (12, 13) all the characteristics of the cooling system can be estimated. This would be count as a paramount finding of the present study.

## Experimental Procedure

**Figure 3:**
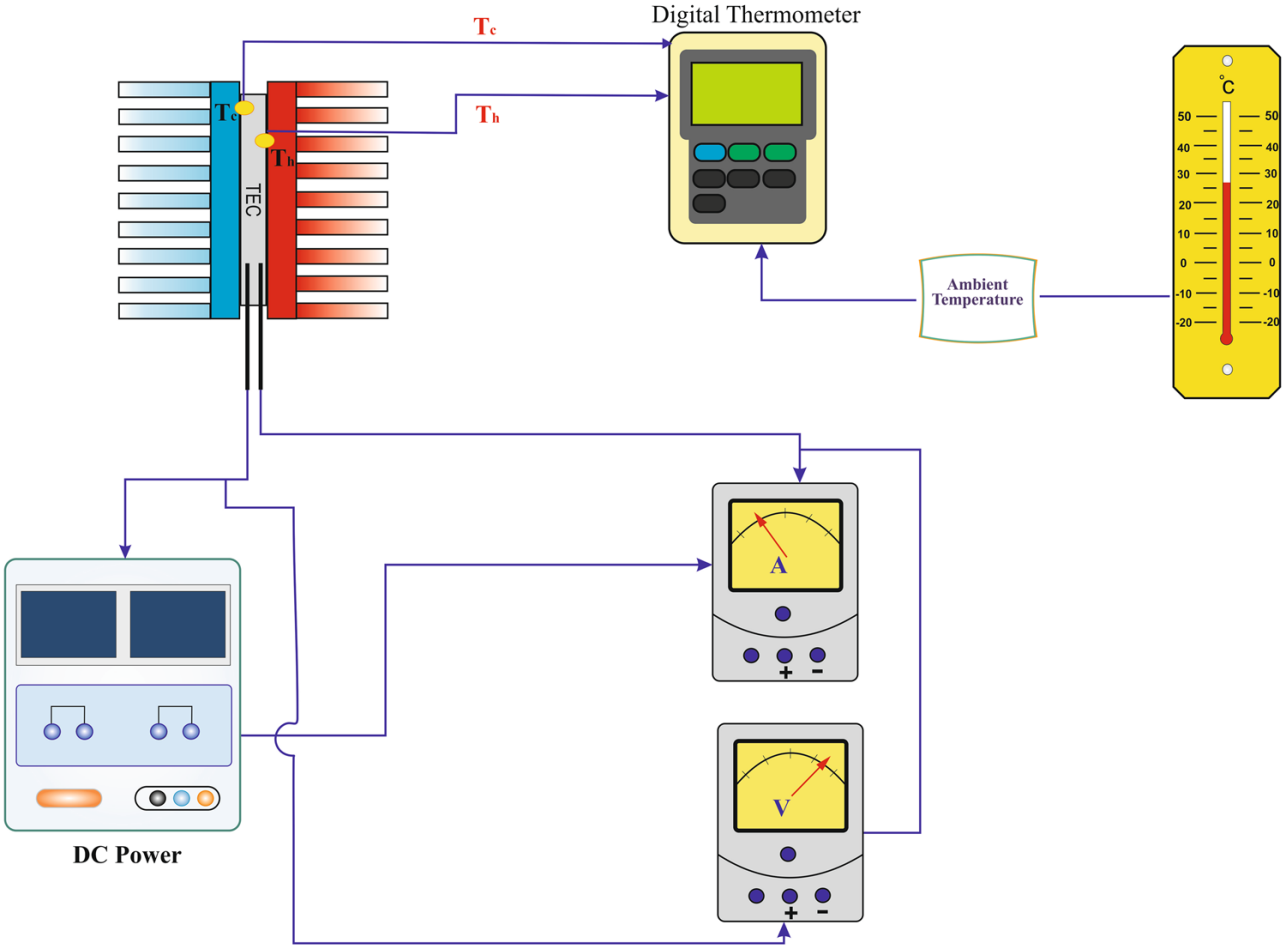
A schematic view of the experimental setup.

A system consists of a thermoelectric module, and two heatsinks were designed, tested, and used to validate the results of mathematical modeling and nonlinear equation (Eq. 16) proposed for the thermoelectric cooling system. Table 1 represented the characteristics of the experimental setup while Fig. 3 shows a pictorial and a schematic view of the experimental system. Table 2 represented the different conditions used in experimental procedures. It should mentioned that the experiments were conducted in three modes. In the first case, the heatsinks are directly placed in the ambient air, and in the latter cases, two fans were used on both sides of the heatsinks operated at 5 *V* and 12 *V* to cool down the system.

**Table 1:** Characteristics of the experimental setup.

Parameter	Value	Unit
**Heatsink**		
Material	Aluminum	
Type of fins	Rectangular	
Thermal conductivity of the wall	167	W/m.K
Dimension	107 mm × 107 mm × 20 mm	
Number of fins	23	
Thermal resistance	1.78	K/W
**Thermoelectric modules**		
Model	TEC1-12704	
Manufacturer	Thermonomic Inc.	
Dimension	40 mm × 40 mm × 3.5 mm	
Seebeck Coefficient	0.04498	V/K
Electrical Resistance	4.36	Ohms
Thermal Conductivity	0.355	W/K
**Data logging**		
Model	BTM-4208SD	
Manufacturer	Lutron Inc	
Analog channels	4	
**Temp. sensor**		
Type K		
Accuracy	±1	°C
**Digital Multimeter**		
Model	DT9205M	
Name	BEST	
DC Current range	0.002–20	A
Current Accuracy	±1.8%	
DC Voltage	0.2–1000	V
Voltage Accuracy	±0.5%

**Table 2:** Different conditions used in experimental procedures.

Case	Condition	Ambient temperature (°C)	TEC cold side temperature (°C)	TEC hot side temperature (°C)	Cold side thermal resistance, R_c_ (°*C* .*W*^−1^)	Hot side thermal resistance, R_h_ (°*C* .*W*^−1^)	Thermoelectric voltage (V)
1	Without fan	25.1	16.1	40.1	6.78	2.61	4.96
2	5V- Fan	25.5	16.6	32.9	1.62	0.67	4.95
3	12V- Fan	24.9	19.7	31.1	0.65	0.44	4.91

## Results and Discussion

As mentioned before, this study aims to propose a new correlation for performance evaluation of a thermoelectric cooler combined with two heatsinks. As mentioned above, an experimental system was designed and tested to validate the results of the new correlation proposed by Eq. 16. Table 3 shows the comparison between the experimental results with mathematical modeling. The results show that mathematical modeling could estimate the behavior of the thermoelectric system within an acceptable accuracy (±12%). There are two reasons for the difference between experimental and mathematical results. First, there is always a numerical error in solving the nonlinear equation (Eq. 16). Second, the thermoelectric properties (Seebeck coefficient, Thermal conductivity, Electrical resistance) are assumed to be constant in the Eq. 16. These properties are a function of temperature.

**Table 3:** Comparison of the experimental results with mathematical modeling.

Case	Condition	heat rate at TEC hot side - Q_h_ (W)	heat rate at TEC cold side - Q_c_ (W)	Experimental current (A)	Mathematical current (A) (Equation 16)	Error (%)
1	Without fan	5.74	1.33	0.89	0.9954	11.8
2	5V- Fan	11.06	5.49	1.05	0.9955	−5.2
3	12V- Fan	14.01	7.96	1.12	0.9866	−11.9

In addition to the above experimental validation, as a case study, another commercial thermoelectric cooler (Model: 9501/127/040 B, manufactured by Ferrotec) has been selected to validate the proposed correlation. Table 4 represents the calculated values of the internal parameters for the selected TEC at the temperature of 50 °C using Eqs. (7–9). To examine the values of electrical current obtained from Eq. 16, the values have been compared with those provided in the TEC’s datasheet, and the results have been presented in Table 5. As can be seen, the minimum and the maximum error is 0.4% and 4.2%, respectively.

**Table 4:** Internal properties of proposed commercial TEC at the temperature of 50 °C.

Property	Symbol	Unit	Value
Seebeck coefficient	*α*	*VK*^*−1*^	0.05999
Electrical resistance	*β*	*Ω*	4.09
Thermal conductivity	*γ*	*WK*^*−1*^	0.44448

**Table 5:** Validation of data for electrical current.

Voltage (V)	I (A)		
	Results of modeling	Catalog values	Error (%)
6	1.25	1.204	−3.8
8	1.66	1.621	−4.2
10	2.078	2.045	−1.6
12	2.488	2.477	−0.4
14	2.897	2.918	0.7
16	3.303	3.368	1.9
18	3.707	3.828	3.1

**Figure 4:**
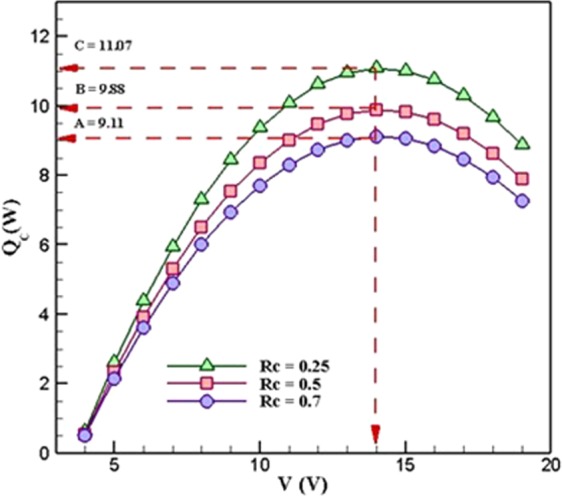
Variations of the cooling power in different values of cold-side thermal resistance when R_h_ = 0.5 *K*.*W*^*−1*^ (TEC Model: Ferrotec 9501/127/040 B).

In order to investigate the effects of changing the values of thermal resistance of the cold-side heatsink, ***R***_***c***_, on the cooling power of the system, the changes of cooling power have been investigated in three different values of thermal resistance (0.25 *K*.*W*^−1^, 0.5 *K*.*W*^−1^, and 0.7 *K*.*W*^−1^) which is shown in Fig. 4. It can be seen that the cooling power of the system increased as the voltage increased. This is continued and hit the highest point at the voltage of 14 *V* for all the values of thermal resistance. After that, it is followed by a considerable decrease in cooling power as the voltage increased. As a result, the maximum cooling power takes place at the voltage of 14 *V* which is the optimum voltage of the system. Moreover, this plot shows that the variations of ***R***_***c***_ have no effect on the optimum voltage of the cooling system. In other words, the optimum voltage in all the three studied ***R***_***c***_ is 14 *V*.

**Figure 5:**
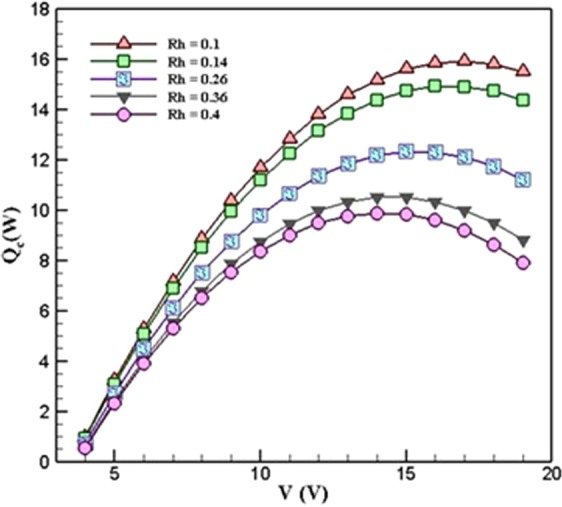
Variation of cooling power with respect to the hot-side thermal resistance, when R_c_ = 0.5 *K*.*W*^*−1*^ (TEC Model: Ferrotec 9501/127/040 B).

**Figure 6:**
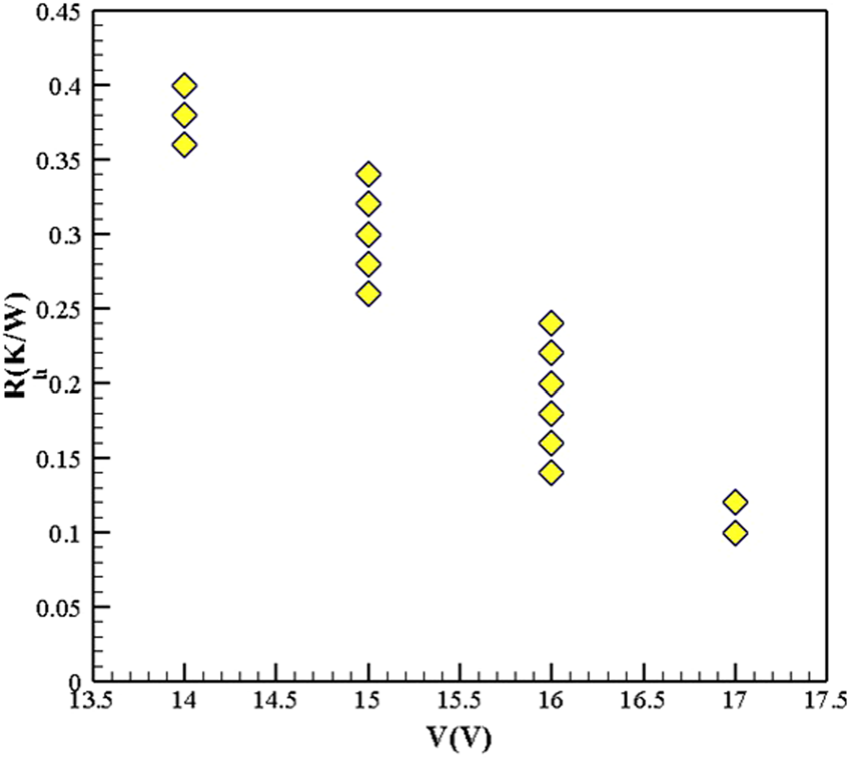
Variations of optimum voltage with respect to the *R*_*h*_ at R_c_ = 0.5 *K*.*W*^*−1*^ (TEC Model: Ferrotec 9501/127/040 B).

On the other hand, in four different values of ***R***_***h***_, the variations of the optimum voltage and cooling power have been investigated, and the results displayed in Fig. 5. The results showed that by decreasing the ***R***_***h***_, the cooling power and optimum voltage increased. Figure 6 shows the effect of changing the ***R***_***h***_ on the optimum voltage at ***R***_***c***_ = 0.5 *KW*^*−1*^. As it is evident, in different ***R***_***h***_, the optimum voltage is different. Thus it is very important for a designer to choose the best values of voltage to have an optimum condition in the TEC cooling system.

**Figure 7:**
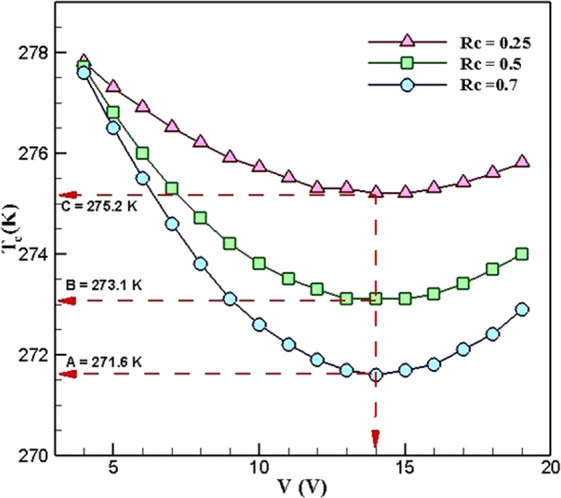
Variations of cold-side temperature in different R_c_, when *R*_*h*_ = 0.4 *K*.*W*^*−1*^ (TEC Model: Ferrotec 9501/127/040 B).

Another important factor in a combination of a heatsink and a thermoelectric cooler is the effects of changing the voltage on the cold-side temperature of TEC. Figure 7 demonstrates the variations of the cold-side temperature of TEC with respect to voltage in different values of the ***R***_***c***_. As can be seen, increasing the ***R***_***c***_ leads to decreasing the cold-side temperature of TEC. On the other hand, the cold-side temperature of TEC decreased as the voltage increased. At the voltage of 14 *V*, which is the optimum voltage of the system, the cold-side temperature of TEC hits the lowest point. Moreover, increasing the voltage to more than 14 *V* leads to increasing this temperature.

**Figure 8:**
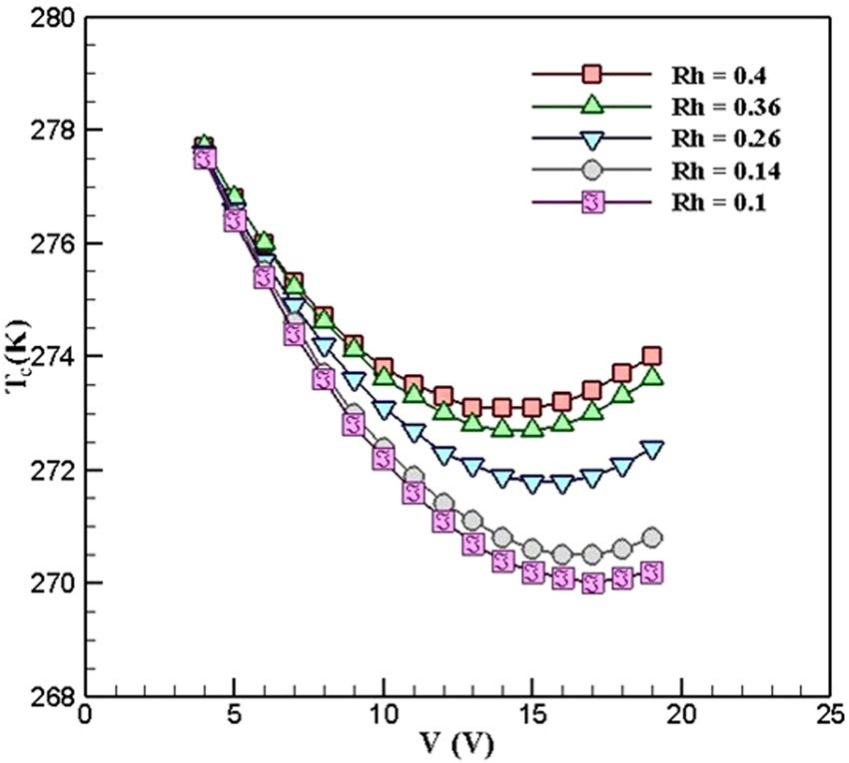
Variations of the cold-side temperature of TEC with respect to voltage in different *R*_*h*_ when *R*_*c*_ = 0.5 *K*.*W*^*−1*^ (TEC Model: Ferrotec 9501/127/040 B).

Figure 8 shows the variations of the cold-side temperature of TEC with respect to voltage in different values of the ***R***_***h***_ when ***R***_***c***_ = 0.5 *K*.*W*^*−1*^. As can be seen, decreasing the value of ***R***_***h***_ leads to decreasing the minimum cold-side temperature of TEC from 273.1 *K* in ***R***_***h***_ = 0.4 *K*.*W*^*−1*^ to 270 *K* in ***R***_***h***_ = 0.1 *K*.*W*^*−1*^. When the ***R***_***h***_ decreases, heat transfer to the ambient takes place easier than before, and this leads to increasing the heat transfer at the cold-side of TEC, which makes cold-side temperature rises.

**Figure 9:**
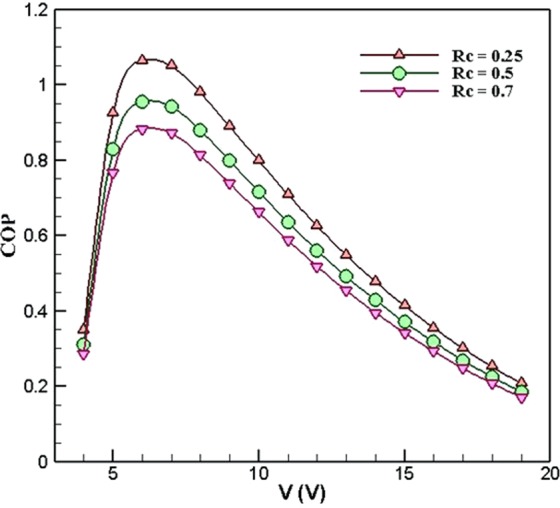
Variations of COP with respect to voltage in different values of *R*_*c*_ when *R*_*h*_ = 0.4 *K*.*W*^*−1*^ (TEC Model: Ferrotec 9501/127/040 B).

Another important factor in every cooling system that should be investigated is the coefficient of performance (COP). Figure 9 displays the variations of COP with respect to voltage in three different values of the ***R***_***c***_ when ***R***_***h***_ = 0.4 *K*.*W*^*−1*^. The results show that decreasing the ***R***_***c***_ from 0.7 *K*.*W*^*−1*^ to 0.25 *K*.*W*^*−1*^, the COP increases. This means that the ***R***_***c***_ has an inverse effect on the COP of the system. The maximum COP of the system was 1.064 which took place at the voltage of 6 *V* when ***R***_***c***_ = 0.25 *K*.*W*^*−1*^. Moreover, in all the cases, the maximum COPs of the system occur in the voltage of 6 *V*, which is different from the optimum voltage (14 *V*) resulted from the maximum cooling power of the system (Fig. 4). This is because of the fact that although at the voltage of 14 *V*, the cooling power (*Qc*) is at its maximum value, the value of the used power is high, which results in decreasing the COP. However, at the voltage of 6 *V*, the cooling power is lower than that of the voltage 14 *V*, but the COP is higher. Thus the optimum voltage depends on the need of the consumers whether the cooling power is important to them or the COP.

**Figure 10:**
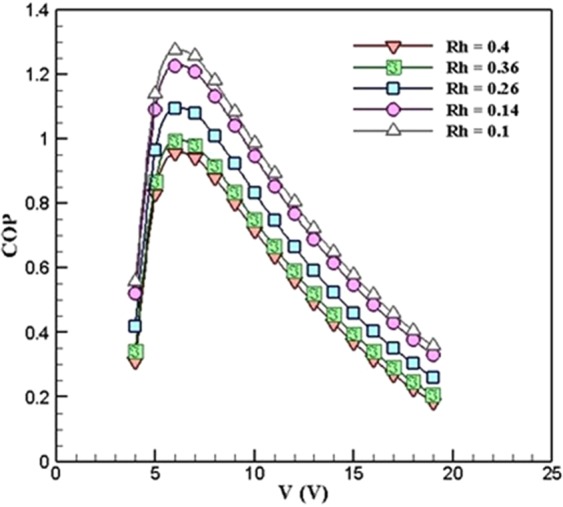
Variations of COP with respect to voltage in different values of *R*_*h*_ when *R*_*c*_ = 0.5 *K*.*W*^*−1*^ (TEC Model: Ferrotec 9501/127/040 B).

Figure 10 shows the variations of COP with respect to voltage in different ***R***_***h***_ when ***R***_***c***_ = 0.5 *K*.*W*^*−1*^. As can be seen, in ***R***_***h***_ = 0.4 *K*.*W*^*−1*^, 0.36 *K*.*W*^*−1*^, 0.26 *K*.*W*^*−1*^, 0.14 *K*.*W*^*−1*^, and 0.1 *K*.*W*^*−1*^, the maximum values of COP is 0.9545, 0.9925, 1.093, 1.226, and 1.273, respectively. Thus as the ***R***_***h***_ decreases, the COP increases. It is interesting to note that in each ***R***_***h***_, the maximum COP takes place at the voltage of 6 *V*. This is due to the fact that by increasing the voltage, the cooling power does not increase to that of electrical power.

The results of Figure 10 shows that both the hot- and cold-side thermal resistance of the heatsinks has an inverse effect on the coefficient of performance of the TEC cooling system.

## Conclusion

The present study was conducted to determine the effects of changing the voltage and the thermal resistance of cold- and hot-side heatsink on the performance of a TEC. For this purpose, the governing equations consisting of conservation of energy and heat transfer have been derived and a third-order nonlinear equation as a function of the internal thermoelectric properties, supplied voltage, heatsink’s thermal resistance, and ambient temperature has been proposed. Solving these equations, the supplied electrical current, COP of the system, and the cooling power can be calculated. As a case study, a commercial TEC has been chosen, and the performance of the system estimated using the proposed equation. The main achievement of this study can be summarized as follows:

- A comparison between the result of modeling and thermoelectric datasheet shows good accuracy of the mathematical modeling.
- The results showed that in a fixed heatsink’s thermal resistance, there is an optimum voltage in which the cooling power of the system is in its maximum value.
- Increasing the thermal resistance of cold-side heatsink leads to decreasing the cold-side temperature of TEC.
- The results showed that both the hot- and cold-side thermal resistance of heatsinks has an inverse effect on the COP of the TEC cooling system.

The represented method in this study can be useful for designers or engineers who are going to use a combination of thermoelectric cooler and heatsinks as a cooling device. The method can assist the researchers in choosing the best value of voltage and system characteristics in a given ambient temperature and heatsinks’ thermal resistance.
